# Small GTPases and Their Regulators: A Leading Road toward Blood Vessel Development in Zebrafish

**DOI:** 10.3390/ijms23094991

**Published:** 2022-04-30

**Authors:** Ritesh Urade, Yan-Hui Chiu, Chien-Chih Chiu, Chang-Yi Wu

**Affiliations:** 1 Department of Biological Sciences, National Sun Yat-sen University, Kaohsiung 804, Taiwan; uraderit@gmail.com (R.U.); paty.121351@gmail.com (Y.-H.C.); 2 Department of Biotechnology, Kaohsiung Medical University, Kaohsiung 807, Taiwan; 3 Doctoral Degree Program in Marine Biotechnology, National Sun Yat-sen University, Kaohsiung 804, Taiwan; 4 Institute of Medical Science and Technology, National Sun Yat-sen University, Kaohsiung 804, Taiwan

**Keywords:** small GTPases, GTP-binding proteins, vascular development and zebrafish

## Abstract

Members of the Ras superfamily have been found to perform several functions leading to the development of eukaryotes. These small GTPases are divided into five major subfamilies, and their regulators can “turn on” and “turn off” signals. Recent studies have shown that this superfamily of proteins has various roles in the process of vascular development, such as vasculogenesis and angiogenesis. Here, we discuss the role of these subfamilies in the development of the vascular system in zebrafish.

## 1. Introduction

**Figure 1:**
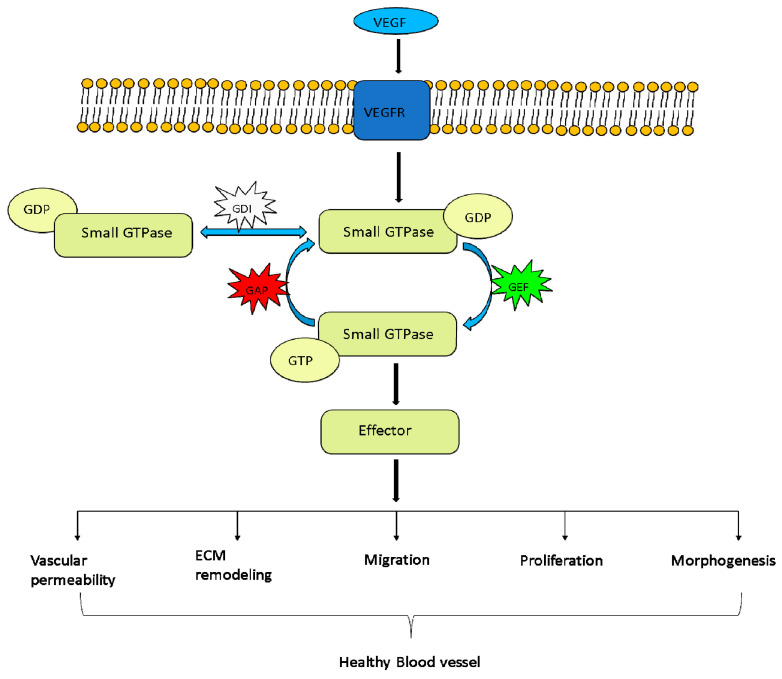
Small GTPase regulation leading to healthy vessels: Cycling of GTPases in the active state and inactive state. Their activation is governed by GEFs, which remove GDP and allow excess cytoplasmic GTP to attach. The binding of active GTPase to effector proteins aggravates the cell response to give rise to blood vessels. GAP, by increasing GTPase activity, turns off the switch for GTPases. Inactive GTPase aggregates in the cytosol via GDIs. By activating effector proteins, downstream processes led to the development of healthy blood vessels (adapted and modified from Cherfils and Zeghouf 2013 [10]).

Small GTPases are GTP-binding proteins frequently found in eukaryotes. These are profoundly reported to have roles in processes such as differentiation, proliferation, morphology, adhesion, survival, migration, apoptosis, cytoskeletal reorganization, cellular polarity, cell cycle progression and many noteworthy biological functions in cells. These proteins cycle between their active form, which is GTP bound, and their inactive form, which is GDP bound, which can affect almost all cellular processes [1]. Approximately 160 members of the small GTPase family have been reported to date [2]. The Ras (rat sarcoma) subfamily of small GTPases contains the largest number of members; hence, sometimes it is called a Ras GTPase [3]. Depending on their structures and functions, these proteins are divided into five main categories: Ras, Ras homology (Rho), Ras proteins in the brain (Rab), Ras nuclear protein (Ran) and adenosine diphosphate ribosylation factor (Arf)/ secretion-associated and Ras-related (Sar) GTPase [4,5]. The regulation of small GTPases (Figure 1) is controlled by three groups of proteins, namely, GTPase-activating/accelerating proteins (GAPs) assisting in hydrolyzing GTP, guanine nucleotide exchange factors (GEFs) stimulating the exchange of GDP to GTP, and guanine nucleotide dissociation inhibitors (GDIs), which accumulate GDP- or GTP-bound small GTPase inside the cytoplasm (by masking their C-terminal isoprenyl group) and terminate its activation. When they are bound to GTP, Ras GTPase forms an association with effectors that lead the way for downstream signaling. These regulators work immediately upstream of the small GTPases to provide a link between small GTPase activation and their receptors [6]. Due to back-and-forth rotation of GTPases, their regulators coordinate and take part in many biological functions. Many regulators of small GTPases coexist in most cells to control the smooth coordination of small GTPases. Many recent studies have revealed that these regulators have their own regulatory mechanism by which they process cellular signals and accumulate specific cell responses. Small GTPases were found to assist the process of blood vessel development. Three pathways are critical for the process of blood vessel development in zebrafish: vascular endothelial growth factor (VEGF) signaling [7], Notch signaling [8] and bone morphogenetic protein (BMP) signaling [9]. Impaired small GTPases can contribute to serious threats such as cancer and developmental malfunctioning [4]. Despite this importance, their regulatory role in vascular development is unclear. Hence, in this review, we discuss them along with their regulators and in blood vessel development in zebrafish.

## 2. Blood Vessel Development

Vasculature development is an important process for the survival of any organism. Despite this much importance, we still are unaware of how the process of its formation takes place. How blood vessels maintain their structure, diameter, permeability, shear stress, etc., these are some of the aspects we are trying to figure out. Some researchers have linked the answers to these questions to endothelial cells, since these cells have the potential to form new vessels with various mechanisms. Several studies on the in vitro culturing of embryonic stem cells (ESCs) showed an endothelial progenitor named hemangioblast [11,12]. Endothelial progenitor cells (EPCs) arise from hemangioblasts, which repair and revascularize the ischemic retina [13]. These EPCs, by two processes, form a blood vessel. The first is vasculogenesis, in which blood vessels form by de novo synthesis [14,15], and the other is angiogenesis, which uses preexisting vessels to extend and form new blood vessels [16]. Angiogenic cues or ischemia increase endothelial permeability, which gives a chance to matrix metalloproteins to debase the extracellular matrix, which relieves endothelial cell (EC)-pericyte contact and ultimately releases growth factors. EC permeability is controlled by various factors, such as thrombin, VEGF and sphingosine 1 phosphate. These factors guide the loss of junctional integrity, which is a reversible process [17]. This gives the bordering cells a space to influx fluids and small molecules due to the absence of cellular contacts. Due to coordinated activation of each GTPase, ECs tend to migrate to promigratory cues and then proliferate to reach their final destination, where they undergo morphogenesis to form a functional lumen and further branches if required [6].

Two forms of angiogenesis have been proposed explicitly: sprouting angiogenesis and intussusceptive angiogenesis. In sprouting angiogenesis, branching of primary blood vessels to form a new vessel takes place [18]. Sequential events are as follows: The desired site directs the ECs, which create a bipolar mode and align endothelial cells and form lumen and tip cells that sprout from distant sites and connect and initiate blood circulation [19]. In intussusceptive angiogenesis, longitudinal splitting of a primary vessel takes place into two different new branches, hence increasing the vascular surface area [20]. Both processes not only provide oxygen but also supply required nutrients to the desired sites and help eliminate waste products. Each angiogenesis is controlled by proangiogenic factors such as VEGF and its receptors VEGFR1 and VEGFR2. Activation of these tyrosine kinase receptors leads to activation of different pathways, such as MAPK, PI3K, and PLCy, favoring angiogenesis [21]. Cancer cells take over some of these molecules to fulfil their own requirements, such as oxygen and nutrients, for metastatic spread. Where insufficient vessels or short growth leads to tissue ischemia, unnecessary vessel growth or abnormal repair can lead to cancer, inflammation disorders, and retinopathy [22]. The process of angiogenesis is governed by activators as well as inhibitors. The mechanism and location of angiogenic activators and inhibitors could lead us to design a specific drug.

## 3. Why Zebrafish and Our Recent Study in the Field of GTPase Related Protein

**Figure 2:**
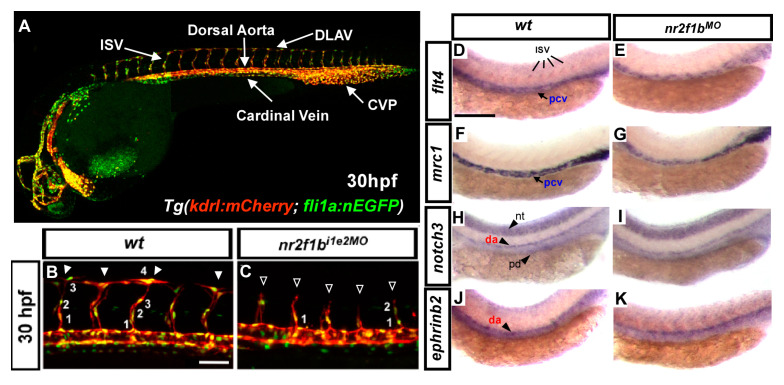
Study the function of genes in vascular development. (**A**) Confocal image of transgenic fish *Tg(kdrl:mCherry; fli1a:nEGFP)* where mCherry expression is in the endothelial cells and GFP expression is in the nucleus at 30 hpf. The image shows clear vessel structures of the dorsal aorta (da), cardinal vein (pcv), ISV, DLAV and CVP. (**B**,**C**) Knockdown of *nr2f1b* in transgenic fish results in vascular defects, i.e., Fewer ISVs migrated to the top of the embryo, and fewer ISV cells migrated per ISV in morphants (hollow arrowheads and fewer numbers) than in the wild-type control (arrowheads). Scale bars in panels (**B**) and (**C**) represent 50 μm. (**D**–**K**) In situ hybridization data showed that knockdown of *nr2f1b* reduced the expression of vascular markers. Scale bars in figures (**D**–**K**) represent 200 μm. Images (**B**–**K**) courtesy of R.-F. Li, reproduced/adapted from Li et al. (2015) [27] with permission from *J. Biomed. Sci*.

Zebrafish (*Danio rerio*) is a freshwater fish belonging to the *Cyprinidae* family native to Southeast Asia [23]. Increasing restriction on using animal model organisms in research has paved the way for zebrafish to become a popular vertebrate model in many fields, such as developmental biology, toxicology and oncology. Zebrafish provide a series of advantages over other vertebrate animal models, such as external fertilization, fecundity, rapid developmental ability, favorable forward and reverse genetic manipulation, availability of cell lines, availability of transgenic lines and tractability to genetic manipulation. Sequencing of the zebrafish genome revealed 70% similarity in its protein coding regions to humans and 84% genes linked with human diseases [24]. Several genetic studies on zebrafish revealed ferocious conservation of molecular pathways in vertebrates for the development and physiology of blood vessels [25]. Using zebrafish to study vascular development has intensively identified many molecules that control artery-vein identity, caudal vein plexus (CVP) formation, and pattern intersegmental vessel (ISV) due to their optical transparency and the availability of labeling techniques for endothelial cells with specific antibodies or tagging with specific fluorescence, allowing us to observe cellular localization, migration, division and rearrangement during vasculogenesis and angiogenesis [20]. Another advantage of the zebrafish is the ability to rapidly and inexpensively downregulate gene expression using morpholino (MO). Morpholinos are oligonucleotides with a modified nondegradable backbone designed to block translation or splicing of a specific mRNA, leading to dramatic reduction of gene expression (Figure 2). 

**Figure 3:**
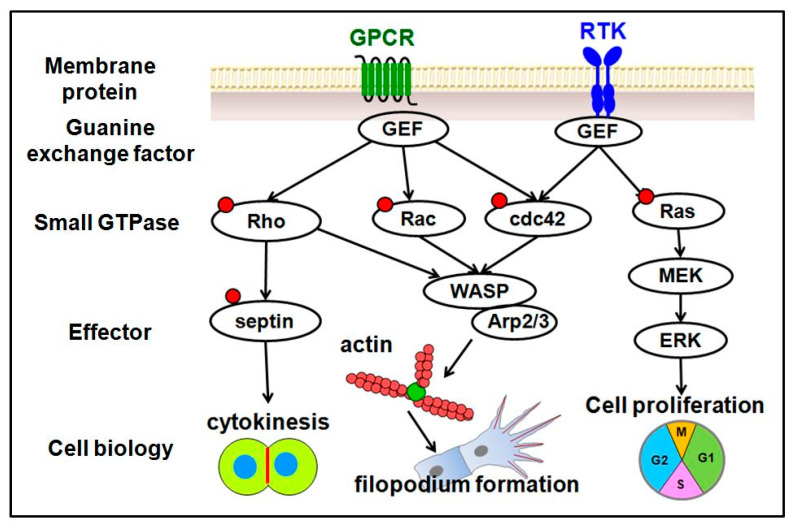
Schematic drawings of the proteins involved in GTPase signals and cellular function related to the cell biological process of vascular development.

We previously reported the role of the transcription factors Islet2 and Nr2f1b in specification of the vein and tip cell identity mediated by the Notch pathway in zebrafish (Figure 2) [26,27]. To further explore this possibility, we used an unbiased microarray approach and identified many novel genes related to vasculature development regulated by the Islet2 and Nr2f1b transcription factors. We noticed an interesting group of GTPase-related genes, including *G-coupled receptor-like (gpcrl), septin 8b (sept8b), Rho-related protein (ect2), rhoub (ras homolog gene family), RAS-like family 11 (rasl11b)* and *Wiskott-Aldrich syndrome protein (WASF1)*. The putative function related to GTPase signals is shown in Figure 3. GTPases are key proteins in many critical biological processes, including hormonal and sensory signals, ribosomal protein synthesis, cytoskeletal organization, signal transduction cascades and motility. Small GTPases are hydrolase enzymes present in the cytosol that can bind and hydrolyze GTP and GDP. These enzymes have been shown to have diverse roles in the development of healthy vasculature. The small GTPase Rap1 has been shown promote VEGFR2 activation and angiogenesis [28]. The Ras GTPase family has been shown to function in vascular patterning via semaphorin-Plexin signaling [29]. However, the GTPase genes we list above do not have any yet known functions in vessels, and we have currently addressed these questions. Since humans and zebrafish share a common mechanism for the process of vessel development [30], we will review available small GTPases and their regulators involved in the process of vascular development.

## 4. Small GTPases and Their Regulators

Due to mutations in the GTPase domain of small GTPase (in various cancers) this family have approximately 160 members, which makes them a superfamily [31]. The process of angiogenesis is controlled by various angiogenic factors, including VEGF. VEGF binding to its tyrosine kinase receptors VEGFR1 and VEGFR2 stimulates downstream signaling cascades such as MAPK, PI3K and PLCγ, which can ultimately contribute to the process of angiogenesis [21]. There is significant evidence supporting the contribution of these proteins as downstream effectors of the VEGF signaling pathway in angiogenic processes. According to their sequence, structure, and functions, this wide-ranging superfamily has been further classified into five subfamilies of Ras, Rho, Ran, Rab, and Arf/Sar GTPase [4,5].

### 4.1. Ras Family

The Ras GTPase family is the first family among others and the most diversified family. Due to prenylation, most members of this family are present in the plasma membrane [32]. The activated Ras members interact with the effector moiety and play a different cellular role in the development, proliferation, differentiation, and survival of eukaryotes [4]. A total of 38 members have been reported in this family [5]. The conserved and ubiquitously distributed forms of the Ras family include H-ras, N-ras, and K-ras, which have different biological functions [31].

Pezeron et al. reported the first cytosolic small GTPase rasl11b in the development of zebrafish and showed that it has a zygotic and maternal origin. Rasl11b’s dorso-marginal expression shows its role in the formation of the endodermal and/or mesodermal layer. Downregulation of rasl11b acts as a suppressor of the EGF-CEF factor *one-eyed pinhead* (oep) phenotype (such as an altered A-P axis, failing to develop endoderm, prechordal plate, and posterior mesoderm [33]) and showed that it can partially rescue prechordal plate and endoderm formation in oep-deficient embryos. However, loss of rasl11b function halted the formation mesendoderm without activation of Nodal signaling when attempted in other than oep mutants. This correlation between oep and rasl11b reveals that oep can influence mesendoderm formation without taking part in the Nodal-Smad2 signaling pathway [34].

Another frequently activated oncogene from the Ras family is K-Ras. Mouse knockout studies have already established their role in normal developmental processes [35,36]. In vivo studies by Liu et al. in zebrafish showed that K-Ras expression starts from the single-cell to throughout the embryo. Morpholino injection showed reduced blood circulation with a lower heart-beat rate, and the accumulation of blood cells was often found away from circulation sites when compared to the negative control morpholino. Apart from these, defects that increased in later stages showed a disorganized subintestinal vein (SIV) with a reduced number of vessel branches along with a reduction in size and/or ectopic blood vessels in K-Ras morpholino-injected embryos. All defects caused by morpholinos could effectively be rescued after coinjection with K-Ras mRNA, supporting its role in hematopoiesis and angiogenesis. Treatment with PI3K/Akt and Mek-Erk1/2 inhibitors provides direct evidence in vivo of the involvement of PI3K-Akt signaling in orchestrating K-Ras signaling for these two salient processes [37].

Semaphorin and its receptors Plexins have been associated with regulating angioblast behaviors [29]. The members of the plexin family co-interact with small GTPases, such as the Rnd, R-Ras, M-Ras and Rap families, and function as Ras-GAPs [38,39,40,41]. In zebrafish, only a single semaphorin3e is expressed in DA, ECs and primary motoneurons and is associated with delayed angioblast migration from DA to structural ISV [29]. Apart from semaphorin, its receptors PlexinD1 and PlexinB2 were found to be expressed in angioblasts. PlxnD1 was found to be expressed in angioblasts and within DA, PCV, and ISV, which shows its involvement in both processes of vasculogenesis and angiogenesis [29]. Loss of one of the receptors, PlxnB2, delayed ISV, which resembles the loss of sema3e morphants, while the loss of another receptor, PlxnD1, in an *out-of-bound* (obd) mutant results in precocious sprouting [42]. This riveting result shows that Sema3e and PlxnD1 do not act as ligand–receptor pairs here for vascular morphogenesis, but PlxnB2 and Sema3e do. A genetic interaction study between PlxnB2 and Sema3e controls the time of sprouting of angioblasts [43]. The transplantation experiment showed that PlxnB2 and Sema3e act autonomously to control the timing of angioblast migration. ECs fail to sense repelling signals produced by semaphorin in the absence of Plexins. Torres et al. morpholino studies in the obd mutant show that loss of one of two Sema3e or PlxnB2 produces an intermediate phenotype, concluding the role of PlxnD1 and Sema3e/PlxnB2 in antagonizing each other’s role in tuning the timing of ISV sprouting but following different signaling and independent pathways downstream of each receptor [43].

Integrins are extracellular matrix receptors present on endothelial cells that play crucial roles in the process of blood vessel development in zebrafish, especially α_5_β_1,_ α_v_β_3_ and α_v_β_8,_ by binding to ECM components [41,44]_._ Lakshmikanthan et al. showed the role of Rap GTPase in the process of angiogenesis for the activation of VEGF signaling and paved the way for angiogenesis. Both isoforms Rap1a and Rap1b are required for the activation of VGFR2 kinase through integrin α_v_β_3._ In zebrafish, Rab1b acts upstream of the VEGF signaling pathway and is expressed in ISV and has a role in initial events in ISV sprouting but does not contribute to vasculogenesis. Combinatorial effects of VEGFR2 inhibitors showed the role of Rap1bs in anterior as well as mid- trunk formation and ISV sensitivity for VEGF signaling [28].

One of the important family members is N-Ras. N-Ras signaling in zebrafish has a high degree of similarity to that in humans and is functionally conserved. N-Ras regulates venous fate of arterial-venous cell specification, hematopoiesis and EC proliferation. Overexpression of N-Ras does not have any impact on hematopoietic markers such as gata1, αe1, pu.1, l-plastin, and mpo, suggesting normal primitive hematopoiesis, although the absence of HSC markers such as cmyb and runx1 proved the complete absence of definitive hematopoiesis. Expression of N-Ras under the lmo2 promoter showed accumulation of blood cells at the axial vessel and heart chamber due to a lack of blood circulation in the head as well as in trunk vessels or could be due to defective cardiovascular development, although embryos did not survive after 5–8 dpf. Injection of fluorescein-coupled latex beads into the atrium proved the involvement of Ras signaling in this disruption of circulation. Apart from all these defects, there was defective assembly of vessels, especially DA or PCV, reduction in ISV length, defective head vasculature and slow heart beating rate in N-Ras embryos compared to control embryos.

As well as small GTPases, GAPs can also have a high impact on blood vessel development. A single allele of Ras GAP called Ras p21 protein activator 1 (RASA 1/p120-RasGAP) was sufficient to cause capillary malformation-arteriovenous malformation (CM-AVM) [45]. GAPs are negative regulators of small GTPase activity. Vascular defects have been noted, although there was no vascular-specific expression of the RASA1 gene. Lack of blood flow to the posterior part, incomplete formation of CVP and large caudal vascular deformities were noted in morphants. Due to this, arterial blood flow had to return to posterior cardinal vain abruptly. RASA1 works as a critical effector downstream of one of the endothelial receptors called the EPHB4 receptor, which promotes the segregation of endothelial cells to form the aorta as well as cardinal vein [46]. A knockdown study found very similar defects in vasculature; in fact, a reduction in RASA1 leads to compromised full function of the EPHB4 receptor. Compared to normal embryos, both morphants (RASA1 and EPBH4) sprouted more venous endothelial cells, and more venous connections were made at the expense of arterial connections. Inactivation of RAS was achieved by RASA1, proving that EPHB4-RASA1-TORC1 signaling could participate in the process of normal blood vessel development. The same phenotypes were noted when another small GTPase called RhebS16H was knocked down [46].

Lamellipodia formation and sprouting of endothelial cells from the ventral part of the dorsal aorta extend toward guiding cues in their environment to orchestrate growing blood vessels. Polo-like kinase 2 (PLK2) is a family protein conserved in ECs of vertebrates that regulates Rap1 activity to control the formation of tip cell lamellipodia but not filopodia and sprouting of endothelial cells. This lamellipodia formation and protrusion during angiogenesis was found to be dependent on focal adhesion kinase and integrin αVβ3 [47]. Knockdown of PLK2 by morpholino reduced sprouting of ECs as well as its migration and overexpression found to overcome these defects. ISVs did not reach DLAV due to failure of migration from the horizontal myoseptum. While doing so, PLK2 makes a contact with PDZ-GEF, a Rap1-GEF, to control the downstream activity of Rap1 to regulate the formation of EC focal adhesion and the growth of lamellipodia to maintain endothelial tip cell behavior.

The Ras family has often been linked to the regulation of neuronal functions. The study conducted by Yeh and Hsu, 2016 showed that members of the Ras family, such as diras1 (diras1a and diras1b), are expressed in the CNS and dorsal neuron ganglion and function in neuronal outgrowth and neuronal proliferation. Wild-type diras1 can elevate or downregulate the members of the Rho family of GTPases, Rac1 and RhoA. A knockdown study by Morpholino proved its involvement in axon guidance and maintaining the numbers of trigeminal ganglions [48].

Rap1b was found to be associated with hematopoietic stem cell development (HSC) development by promoting Notch signaling. Rap1b promotes specification of posterior lateral plate mesoderm (PLPMs) by encouraging notch signaling. However, while migrating to midline, fibronectin directs the PLPMs along the somite boundary via integrin β1. Rap1b induces the spreading, migration and adhesion of PLPMs to somites to stimulate HE specification. Rap1b was not found to be involved in the process of vascular development but was critical for HSC development. Rap1b is ubiquitously expressed and promotes HSC development by inducing hemogenic endothelium (HE) development in a cell autonomous manner [49].

### 4.2. Rho Family

This family, along with its regulators, controls various cellular processes, including cell polarity, cell proliferation, membrane transport, apoptosis, gene expression, and membrane transport [50,51]. Recently, the role of these small GTPases in the process of angiogenesis was reviewed by Bryan and D’Amore, 2007 [6]. The Rho family is an essential downstream effector of VEGF signaling that induces angiogenic development. Most studies of this family are associated with RhoA, Rac1 and Cdc42. Regulators of this family control various biological activities via activation or deactivation of small GTPases. The Rho family downstream of the VEGF receptor transmits various signals to activate MAPK, PI3K and PLCγ, which are the main signaling pathways that take place during the process of blood vessel development [21].

Vascular permeability is coordinated by loosening and creating a space between the cells to facilitate the influx of macromolecules. Rho GTPase was found to increase vascular ECs permeably, destabilizing adherens and tight junctions. The cell–cell contact junctions of ECs contain Rac1 and Cdc42, and these junctions dissociate during an increase in permeability [52]. RhoC negatively regulates vascular permeability in a VEGF-dependent manner by compensating for EC loss. It maintains homeostasis by creating a balance between vascular injury and repair. Apart from this, it prevents acute endothelial hyperpermeability in zebrafish. RhoC was found to be expressed in DA, PCV, ISVs and NT. However, when injected with morpholinos, no vascular defects were observed [53].

Remodeling and degradation of ECM pave the way for EC to proliferate by following angiogenic cues such as VEGF in the surroundings in the absence of cell–cell contacts to build a functional lumen. The interaction between Arhgap29, a RhoA-GAP, and its binding partner Ras interacting protein 1 (Rasip1) is necessary to modulate EC polarity and cell adhesion to the ECM to activate RhoA signaling to orchestrate the lumen [54]. RhoA’s role in a study conducted by Zhu et al. showed its importance in embryonic survival [55]. The ubiquitous expression of RhoA during early embryogenesis and reduction in the level of RhoA can lead to shrinkage in overall body size along with reduced head size and body length [56]. These defects could be due to increases in the level of apoptosis during embryonic development. As a consequence, there is a reduction in two crucial factors: one is the reduction in the activation of Erk, a growth-promoting factor, and the reduction in bcl-2, an anti-apoptotic factor that could be due to an increase in apoptosis. Regulation of cell survival by RhoA is achieved via the Mek/Erk pathway during embryonic development [55]. Depletion of the RhoA-GAP called Arhgap29 increased RhoA GTPase signaling but repressed Cdc42 and Rac1 GTPase signaling. 

Filopodia are thin finger-like protrusions present on the leading edge of ECs that sense their microenvironment and direct the tip EC toward promigratory signals. On the other end of the EC, adhesion should be released for the forward movement of EC. Several studies have shown that Cdc42 is associated with the formation of filopodia [57,58,59]. A recent study showed that these filopodia drive angiogenesis in response to activation of Cdc42 [58,59]. Ventral migration of these filopodia from caudal vein primordia leads to CVP formation. Filopodia are filled with linear F-actin filaments for CVP formation. Bmp signaling has been shown to be responsible for the migration of ECs toward the ventral side independent of EC fate determination. Given that Cdc42 regulates EC morphology, motility, proliferation and survival, this could regulate BMP signaling to bring about normal CVP formation. GAPs are negative regulators of tip cell angiogenesis, and they limit proangiogenic factors to stabilize the vasculature. One of the Rho-GAPs called ARHGAP18 was found to have a role as a fine tuner for vascular morphogenesis. It is an endogenous molecule that is expressed in ECs and curbs the formation of tip cells to promote junctional integrity [60,61]. It acts on Rho-C to destabilize EC junctions in a ROCK-dependent manner. When it is knocked down by morpholino, increased ISV lengths may be due to an increase in filopodia, supporting its role in hypersprouting [60]. It would be interesting to determine how these factors contribute to VEGF-mediated angiogenesis. Arhgef9b and fgd5 are the Cdc42 GEFs expressed in zebrafish. Apparently, Arhgef9b reduced the number of sprouts from caudal vein primordia and filopodia were noted, which shows that Arhgef9b but not fgd5 could act as a Cdc42-GEF to regulate Bmp signaling to form CVP [59]. The role of Cdc42 along with transporter proteins has been associated with the normal eye development and survival of cells in the eye [57]. However, Cdc42 inhibition severely reduces the speed at which ISVs are formed, and this reduction could be correlated with the reduction in the formation of filopodia and defects in EC proliferation; hence, inappropriate formation of tip cells occurred. Given that Cdc42 regulates the sprouting of EC to form ISVs, it would not be wrong to call it a positive regulator of vessel sprouting. Similar effects have been observed during retinal angiogenesis, showing that similar pathways are followed for vessel development in these two organs [58]. While orchestrating the patterns of vessels, RhoA GEF Syx interacts with angiomotin in the presence of VEGF-A to regulate EC migration [62,63]. A recent study showed that these two interact with a scaffold protein and form a ternary complex to promote the migration of endothelial cells [64]. Coordination between these scaffold protein is require to activate and regulate RhoA activity to lead the tip cell toward guiding cues. Wu et al., 2011 reported that Syx and RhoA regulate not only cell junctions but also EC directional migration by forming lamellipodia [65]. RhoA and Syx show localization in the gradient-dependent manner of VEGF-A toward the leading edge. Cotrafficking of RhoA and Syx is required for cell migration, which depends on another family of small GTPases Rab GTPase, showing that they work interdependently to maintain structure of embryos [65].

βPix is a scaffold protein, and a GEF for Rac and Cdc42 binds to p21-activated kinase (Pak) to regulate vascular stability. It is expressed in embryonic development in the brain as well as large blood vessels. It mainly contributes to embryonic vascular stability and hydrocephalus. Pak2a signaling works downstream of βPix to regulate cerebrovascular development. Loss of βPix led to hemorrhage in the head, signifying its part in cerebral vessel stability instead of vessel-specific breakage. Mutants were found to develop hydrocephalus [2]. Another study conducted by Liu et al. showed that βPix binds to an ARF-GAP called G-protein coupled receptor kinase interacting target (Git1); hence, Git1 functions as a molecular link between integrins and βPix, bringing about a stable vascular system [41]. The complex formed by βPix, integrin α_v_β_8_ and Git1 regulates not only vascular stability but also endothelial cell proliferation and cerebral angiogenesis [41].

Engulfment and cell motility 1 (ELMO1) and dedicator of cytokinesis 180 (DOCK1) form an ELMO1/DOCK1 complex and work as a bipartite GEF to regulate monomeric GTPase Rac1 activity [66]. ELMO1 expressed in different developmental stages of embryogenesis is required for the formation of functional DA, PCV and ISVs, while DOCK180 is expressed predominantly in DA and PCV. Rac has previously been associated with embryonic vascular development [67] and is expressed ubiquitously throughout embryogenesis [68]. The ELMO1/DOCK1 complex works downstream of Netrin-1 (an axonal guiding molecule) and interacts with one of the endothelial receptors called the Unc5B receptor to specifically activate Rac1 to achieve vessel formation. Activation of Rac1 GTPase solely depends on ELMO1; without ELMO1, Rac1 is not activated [69]. ELMO1 found in DA and PCV activate vascular Rac1 to lead the migrating cell toward DLAV. ELMO1/DOCK1 in vitro data apparently did not support its role in VEGF-induced activation of Rac1 for the sprouting of ECs. Overexpression of ELM1 and DOCK1 reduced the total number of apoptotic endothelial cells, which encouraged blood vessel development and EC survival during embryonic development. This protection of ECs from apoptosis was achieved by the reduction in the number of caspase 3/7 molecules via activation of PI3K/AKT signaling to facilitate proper development of functional blood vessels [66]. These findings support the spatiotemporal activation of Rac GTPase by its GEF to bring about functional and healthy blood vessels.

Vascular pruning is a process of removing redundant vessels that form during early vascular growth by the process of apoptosis to form a normal vascular system. It is a crucial process to bring normal functional vasculature. FYVE, Rho-GEF, and PH domain–containing 5 (FGD5) is a Rho-GEF that is expressed in endothelial progenitors as well as mature ECs and regulates the function of Cdc42 small GTPase in both mice and zebrafish [70]. The expression of FGD5 is predominantly achieved in the endothelial lining of large blood vessels, such as DA, ISVs and PCV. To activate Cdc42, FGD5 binds to Cdc42 and activates Hey1-p53-mediated apoptosis in ECs. Overexpression of FGD5 leads to a reduction in the levels of RhoA, and Rac1 shows an indirect downstream relationship between them. Overexpression of FGD5 leads to activation of the Notch signaling pathway by Cdc42 via the MAPK kinase pathway. Hence, FGD5 could be the factor responsible for the aging and survival of vasculature [70].

In sprouting, one cell from the quiescent stage migrates and extends its filopodia toward guiding cues to the dorsal side from the ventral part of the DA to become a leading tip cell. This tip cell expresses genes such as dll4, flk-1 and flk-4 and suppresses bottom cells to become tip cells. GAPs are negative regulators of tip cell angiogenesis by limting proangiogenic factors to stabilize the vasculature. 

Rho signaling has been elucidated in the regulation of atrioventricular canal (AV) and cardiac looping. RhoU is expressed in the atrioventricular canal (when it forms) and regulates cell adhesion molecules (such as N-cadherin and alcama) between cardiomyocytes through the Arhgef7/kinase pathway. Highly conserved RhoU in vertebrates was found to have gene duplication in zebrafish, resulting in Rhoua and Rhoub. Wnt signaling may regulate the expression of this atypical Rho-GTPase. RhoU/Arhgef7/Pak signaling drives the formation of cell junctions between cardiomyocytes and promotes cell–cell adhesion [71] and shapes the cells to bring a functional heart. To maintain cell-adhesion molecules, RhoU effectors such as Arhgef7 and Pak must be maintained for the functioning of AV cardiomyocyte cell junctions. RhoU primarily functions to change the shape of AV cardiomyocytes but does not necessarily affect their fate specification and patterning [71].

### 4.3. Rab Family

Rab-GTPases have a role in cell directional migration via endocytosis and trafficking [72]. The binding of VEGF-A to its receptor VEGFR2 triggers the endocytosis of transmembrane receptors by Rab13. Rab13 mRNA was found to be expressed in the vessels of the trunk [73]. Rab13 GTPase associates with Syx (a RhoA-GEF) at the leading edge of the tip cell. Depletion of Rab13 hampered the sprouting of ISVs and weakened the directionality of tip cells. Rab13 mediates tight junction recycling between the trans-Golgi network and recycling endosomes. VEGF guides Rab13 to direct cell migration. Knockdown of Rab13 not only reduced ISV length but also distorted the shape of tip cells, confirming its role in directional migration. There were no defects reported in DA, which shows its specificity for angiogenesis but not for vasculogenesis [65].

Rab4a and Rab4b have been found to regulate the endocytosis of VEGFR2 trafficking and signaling during the migration and proliferation of endothelial cells. In vitro data show that in early endosomes, VEGFR2 is coexpressed with Rab4a but not Rab11a. GDP-Rab4a increased the level of VEGFR2 in endosomes. Reduction in Rab4a increased intracellular VEGF-A, and its intracellular signaling resulted in increased endothelial cell proliferation. VEGF-A-induced endothelial cell migration is inhibited when Rab4a or Rab11a is reduced. Rab4a and Rab11a are both essential for the development of endothelial tubules and are required for the formation of blood vessels. Depletion of Rab4a in zebrafish caused defects in the formation of both ISVs and DLAV, apart from the fact that ISVs are often missing and terminate before they mature completely. Reduction in either Rab4a or Rab11a has morphological, developmental and detrimental effects [74].

Rab11 signaling has been shown to be involved in lumen formation in the gut of zebrafish. The formation of the lumen takes place through different processes, and membrane trafficking is one of them. In zebrafish, during gut development, multiple small lumens are formed that merge and form a single continuous lumen [75]. This single, continuous lumen formation takes place via Rab11-mediated signaling in the gut of zebrafish. Rab11a regulates the recycling of basolateral and apical membrane proteins, which is a critical step during lumen resolution to form a single continuous lumen [75]. Rab5 was found to be associated with nodal signaling in early embryonic development. Out of four orthologs, Rab5a is teleost-specific and is expressed in medaka. All four orthologs were mostly found to be expressed in the head region (brain). A Morpholino study showed that Rab5ab is involved in regulating nodal signaling [76].

VEGFR2 endocytosis requires the activation of Rab5A/Rab4A by being in the GTP-bound state to develop into zebrafish embryos. Physical interaction between the transporter protein (Sec14l3/SEC14L2), VEGFR2 and Rab5A/Rab4A leads to activation of VEGFR2 signaling by regulating angioblasts and venous progenitors to develop arteries and veins [77].

A balance in the endocytic trafficking of Rab5c is vital for the specification and production of hematopoietic stem and progenitor cells (HSPCs) [78]. Rab5c regulates endocytic trafficking of Notch ligand and its receptor for the cell fate transition from ECs to hemogenic endothelium (HE). Downregulation or overexpression of Rab5c led to HE specification, production, survival defects and HSPC development (via Notch signaling followed by Akt signaling for HE specification). Rab5c is highly expressed in the ventral wall of DA (VDA), a part where HE specification takes place and is restricted to definitive hematopoietic tissues; hence, it is speculated that it participates in the development of HSPCs [78]. A recent study has shown that Rab5c prevents the degradation of VEGFR2 in order to restore tip cell identity and control gene expression of VEGF target genes [79]. 

### 4.4. Arf Family

ADP-ribosylation factor-like 6 (Arl6) is a small GTPase that functions in cellular signaling and protein and membrane transport [80]. Arl6 interacts with another maternally expressed protein called Arl6 interacting protein (Arl6ip). Arl6ip was found to be expressed in various organs (for other organs, refer to [81]) and in the trunk of zebrafish. Knockdown of this particular protein (Arl6ip) showed defects in trunk formation suspected to have a role in heart development along with other organs [81].

Chen et al. showed a different role of Arf5. An organic contaminant called trimethyltin chloride (TMT) induces vascular toxicity, including a reduction in the distance between ISVs, leading to an overall reduction in body length. Arf5 is necessary and plays a significant role in inducing TMT-induced vascular deformities [82].

ArfGAP with a dual PH domain 2 (ADAP2) with GAP activity for Arf6 has a role in heart development. Knockdown of ADAP2 results in blood circulation defects and curved tails. The maternally and zygotically expressing ADAP2 is present in the heart and the region corresponding to the bulbus arteriosus in zebrafish [83]. Arf-GAP, called G protein-coupled receptor kinase interacting target (GIT1), interacts with Rho family GEF βpix (especially Rac and Cdc42) to stabilize blood vessels. Interaction between GIT1 and βpix with integrins regulates vascular stability, endothelial cell proliferation and cerebral angiogenesis. GIT1 is ubiquitously expressed in zebrafish, and its knockdown leads to an increase in hemorrhage, proving its role in vascular stabilization [41].

Apart from growth factors, integrins have been implicated in the process of angiogenesis. Brag2 is recognized as an Arf-GEF for Arf4, Arf5, and Arf6. An in vitro study showed its role in angiogenic sprouting, migration and adhesion of ECs. In vivo experimental silencing of Brag2 showed vascular and developmental defects in zebrafish. Silencing of Brag2 leads to defects mostly related to the formation of DLAV, ISVs and parachordal lymphangioblasts (PL-lymphatic system precursor), showing its role in vascular patterning and stability. Knockdown of both orthologs of Brag2 leads to severe defects in DLAV, ISVs and sometimes the absence of PL. Brag2-mediated activation of Arf5 and Arf6 leads to developmental and pathological angiogenic sprouting of ECs through regulation of adhesion mediated by β1- and β3-integrins [84].

Golgi brefeldin A-resistant factor 1 (Gbf1) is a maternally and zygotically expressed high molecular weight GEF for the Arf-GTPase family that regulates organelle structure and vesicle trafficking. Gbf1 is ubiquitously expressed in the early stage, but it was later found to be expressed in the head region. Isolation of cells showed its expression in ECs to develop vasculature in a cell autonomous manner. Mutated form of this specific GEF fail to activate Arf1 and are unable to recruit cargo complex COPI. A zebrafish mutant line was created by using the mutagen N-ethyl-N-nitrosourea (ENU), which carries the T→G transition on the 23rd exon of the Gbf1 locus. The mutant embryo displayed hemorrhage in the trunk and head regions. Mutants showed pigmentation reduction in the head region and short caudal fins in Mendelian inheritance. Blood cells leak into the head, eye and trunk, which leads to the death of an embryo within 96 hpf. Intracerebral vessels in the head and ISV in the trunk were broken or sometimes disappeared or disconnected, resulting in dissociation of ECs, which could be due to disruption of vascular integrity or homeostasis in mutants [85].

Brefeldin A inhibited guanine nucleotide exchange 1 and 2 (BIG1 & BIG2) protein 1 (arfgef1 and arfgef2 homolog in zebrafish) and is the GEF for two small GTPases, Arf1 and Arf2. Both GEEs are ubiquitously expressed in zebrafish. Knockdown of either BIG1 or BIG2 in zebrafish was associated with EC migration during blood vessel formation. An *in vitro* study showed their involvement in the process of capillary tubule formation and EC migration by modulating actin cytoskeleton organization in HUVECs. Knockdown of BIG2 interferes with the completion of ISVs without reflecting on its numbers, and a reduction in PCV width was observed during embryonic development. BIG1 and BIG2 reduction suppressed the expression level of VEGF and EC migration in the process of blood vessel development [86].

## 5. Conclusions

These studies have shown that Ras superfamily of proteins has importance in many processes that are sufficient to develop completely functional and healthy vessels to carry different nutrients and macromolecules to the entire body. Future studies are still necessary to decode and stage the specific role of Ras-GTPases to fill the gap in vessel development. Their functional role in blood vessel development could guide us to form therapeutic strategies for diseases related to vascular development.

## Abbreviations

ISV, intersegmental vessel; CVP, caudal vein plexus; DA, dorsal aorta; PCV, posterior cardinal vein; DLAV, dorsal longitudinal anastomotic vessel; SIV, subintestinal vein; GAP, GTPase-activating protein; GEF, guanine nucleotide exchange factor; GDI, guanine nucleotide dissociation inhibitor; VEGF, vascular endothelial growth factor; BMP, bone morphogenetic protein; EC, endothelial cell.
